# In Vitro and In Silico Approaches for the Evaluation of Antimicrobial Activity, Time-Kill Kinetics, and Anti-Biofilm Potential of Thymoquinone (2-Methyl-5-propan-2-ylcyclohexa-2,5-diene-1,4-dione) against Selected Human Pathogens

**DOI:** 10.3390/antibiotics11010079

**Published:** 2022-01-10

**Authors:** Kamal A. Qureshi, Mahrukh Imtiaz, Adil Parvez, Pankaj K. Rai, Mariusz Jaremko, Abdul-Hamid Emwas, Avinash D. Bholay, Muhammad Qaiser Fatmi

**Affiliations:** 1 Department of Pharmaceutics, Unaizah College of Pharmacy, Qassim University, Unaizah 51911, Saudi Arabia; 2 Department of Biosciences, COMSATS University Islamabad, Islamabad 45600, Pakistan; mahrukhimtiaz92@yahoo.com; 3 Department of Biotechnology, School of Chemical and Life Sciences, Jamia Hamdard University, New Delhi 110062, India; adilparvez.92@gmail.com; 4 Department of Biotechnology, Faculty of Biosciences, Invertis University, Bareilly 243123, India; pankaj.r@invertis.org; 5 Smart-Health Initiative (SHI) and Red Sea Research Center (RSRC), Division of Biological and Environmental Sciences and Engineering (BESE), King Abdullah University of Science and Technology (KAUST), Thuwal 23955, Saudi Arabia; mariusz.jaremko@kaust.edu.sa; 6 Core Labs, King Abdullah University of Science and Technology (KAUST), Thuwal 23955, Saudi Arabia; abdelhamid.emwas@kaust.edu.sa; 7 Department of Microbiology, KTHM College, Savitribai Phule Pune University (SPPU), Nashik 422002, India; avinashbholay@kthmcollege.ac.in

**Keywords:** antimicrobial activity, anti-biofilm activity, molecular docking, molecular dynamics simulations, time-kill kinetics, thymoquinone

## Abstract

Thymoquinone (2-methyl-5-propan-2-ylcyclohexa-2,5-diene-1,4-dione; TQ), a principal bioactive phytoconstituent of *Nigella sativa* essential oil, has been reported to have high antimicrobial potential. Thus, the current study evaluated TQ’s antimicrobial potential against a range of selected human pathogens using in vitro assays, including time-kill kinetics and anti-biofilm activity. In silico molecular docking of TQ against several antimicrobial target proteins and a detailed intermolecular interaction analysis was performed, including binding energies and docking feasibility. Of the tested bacteria and fungi, *S. epidermidis* ATCC 12228 and *Candida albicans* ATCC 10231 were the most susceptible to TQ, with 50.3 ± 0.3 mm and 21.1 ± 0.1 mm zones of inhibition, respectively. Minimum inhibitory concentration (MIC) values of TQ are in the range of 12.5–50 µg/mL, while minimum biocidal concentration (MBC) values are in the range of 25–100 µg/mL against the tested organisms. Time-kill kinetics of TQ revealed that the killing time for the tested bacteria is in the range of 1–6 h with the MBC of TQ. Anti-biofilm activity results demonstrate that the minimum biofilm inhibitory concentration (MBIC) values of TQ are in the range of 25–50 µg/mL, while the minimum biofilm eradication concentration (MBEC) values are in the range of 25–100 µg/mL, for the tested bacteria. In silico molecular docking studies revealed four preferred antibacterial and antifungal target proteins for TQ: D-alanyl-D-alanine synthetase (Ddl) from *Thermus thermophilus*, transcriptional regulator qacR from *Staphylococcus aureus*, N-myristoyltransferase from *Candida albicans*, and NADPH-dependent D-xylose reductase from *Candida tenuis.* In contrast, the nitroreductase family protein from *Bacillus cereus* and spore coat polysaccharide biosynthesis protein from *Bacillus subtilis* and UDP-N-acetylglucosamine pyrophosphorylase from *Aspergillus fumigatus* are the least preferred antibacterial and antifungal target proteins for TQ, respectively. Molecular dynamics (MD) simulations revealed that TQ could bind to all four target proteins, with Ddl and NADPH-dependent D-xylose reductase being the most efficient. Our findings corroborate TQ’s high antimicrobial potential, suggesting it may be a promising drug candidate for multi-drug resistant (MDR) pathogens, notably Gram-positive bacteria and *Candida albicans.*

## 1. Introduction

Hospital-acquired infections caused by multi-drug resistant (MDR) pathogens are among the leading causes of mortality in hospitalized patients [1,2,3,4,5]. These pathogens include methicillin-resistant *Staphylococcus aureus* (MRSA), vancomycin-resistant *Staphylococcus aureus* (VRSA), vancomycin-resistant *Enterococci* (VRE), and extended-spectrum beta-lactamase (ESBL) producing organisms, including *Escherichia coli* (*E. coli*) *Pseudomonas aeruginosa* (*P. aerugenosa*), *Acinetobacter baumannii* (*A. baumannii*), *Klebsiella pneumoniae* (*K. pneumoniae*), *Klebsiella oxytoca* (*K. oxytoca*), *Proteus mirabilis* (*P. mirabilis*), *Salmonella enterica* (*S. enterica*), *Neisseria gonorrhoeae* (*N. gonorrhoeae*), *Haemophilus influenzae* (*H. influenzae*), *Kluyvera* species, and *Enterobacter aerogenes* (*E. aerogenes*). This list includes second-and third-generation cephalosporins, i.e., cefaclor, cefoxitin, cefuroxime, cefixime, cefotaxime, cefpodoxime, ceftazidime, and ceftriaxone; fluoroquinolones, i.e., levofloxacin, ciprofloxacin, moxifloxacin, ofloxacin, gemifloxacin, and delafloxacin; penicillin with a beta-lactamase inhibitor; carbapenems, i.e., imipenem-cilastatin, meropenem, ertapenem, doripenem, panipenem-betamipron, and biapenem [6]. The treatment of antibiotic-resistant hospital-acquired infection is a difficult challenge for public healthcare. Therefore, the discovery of novel antimicrobial drugs and drug targets is necessary to combat potentially fatal MDR infections [1]. 

Plants produce several secondary metabolites to combat environmental pathogens [4,5]. *Nigella sativa*, a well-known and invaluable plant-grows naturally in southern Europe, northern Africa, and southern Asia. Traditionally, the seeds and leaves of this plant have been used as a culinary spice in Asian and Middle Eastern cuisines [7]. Thymoquinone (2-methyl-5-propan-2-ylcyclohexa-2,5-diene-1,4-dione; TQ), a principal bioactive phytoconstituent of *Nigella sativa* essential oil, exhibits a wide range of potential medicinal properties [7]. Many reports suggested that TQ possesses a range of bioactive properties, including antimicrobial, antiviral, antiparasitic, anticancer, and anti-inflammatory effects [7,8,9,10,11,12]. 

Thus, the current study aimed to investigate TQ’s antimicrobial potential, time-kill kinetics, and anti-biofilm activity against a range of selected human pathogens, including 10 Gram-positive bacteria, seven Gram-negative bacteria, and two fungal strains. This study also investigates TQ’s molecular interactions with various enzymes retrieved from bacterial and fungal pathogens through in silico molecular dockings of TQ with various antimicrobial target proteins. Furthermore, we evaluated the structural and thermodynamic properties of the four best TQ-enzyme complexes using 100 ns long molecular dynamics (MD) simulations. 

## 2. Results 

**Figure 1:**
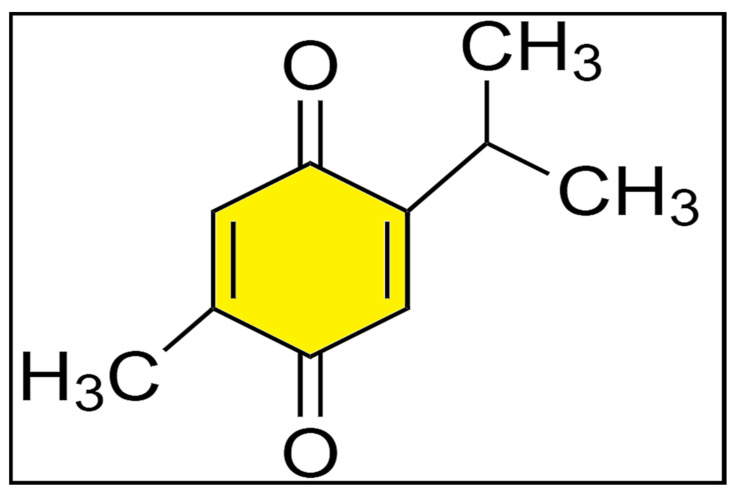
The structure of TQ.

Fourier-transform infrared spectroscopy (FT-IR) analysis revealed that the procured TQ sample exhibited a similar spectroscopic pattern to standard TQ (Figures S1 and S2), as expected. The structure of TQ is given in Figure 1.

### 2.1. Antimicrobial Susceptibility Screening

Antimicrobial susceptibility screening revealed that the clinical isolates are multi-drug resistant and confirmed that they are MRSA (Supplementary Materials Table S1).

### 2.2. Preliminary Antimicrobial Activity

**Figure 2:**
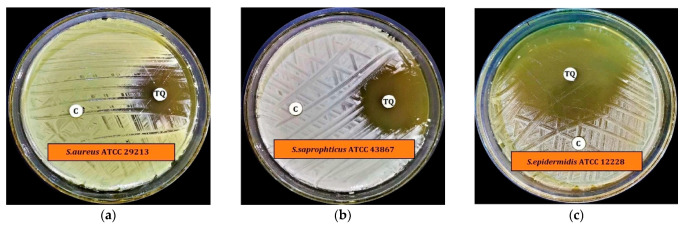

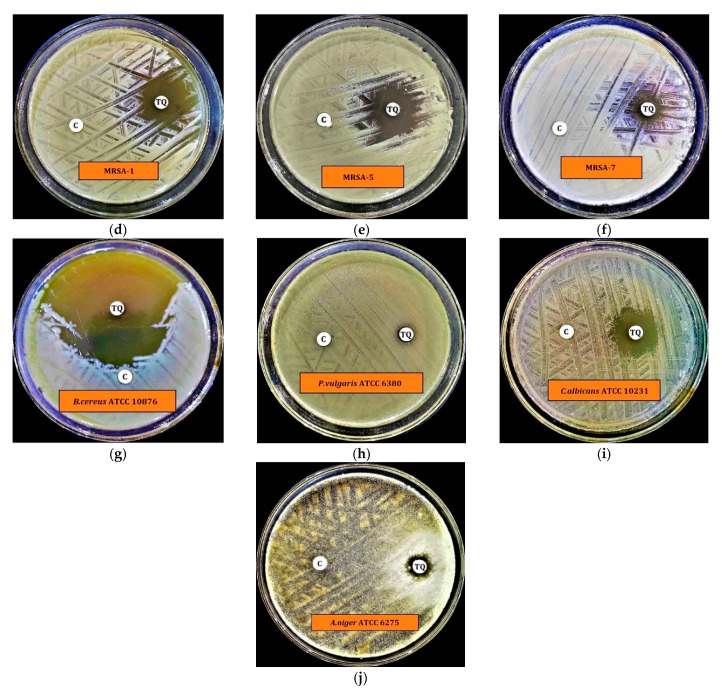
Preliminary antimicrobial activity of TQ against selected human pathogens; (**a**) *S. aureus* ATCC 29213, (**b**) *S. saprophyticus* ATCC 43867, (**c**) *S. epidermidis* ATCC 12228, (**d**) MRSA-1, (**e**) MRSA-5, (**f**) MRSA-7, (**g**) *B. cereus* ATCC 10876, (**h**) *P. vulgaris* ATCC 6380, (**i**) *C. albicans* ATCC 10231, (**j**) *A. niger* ATCC 6275.

**Figure 3:**
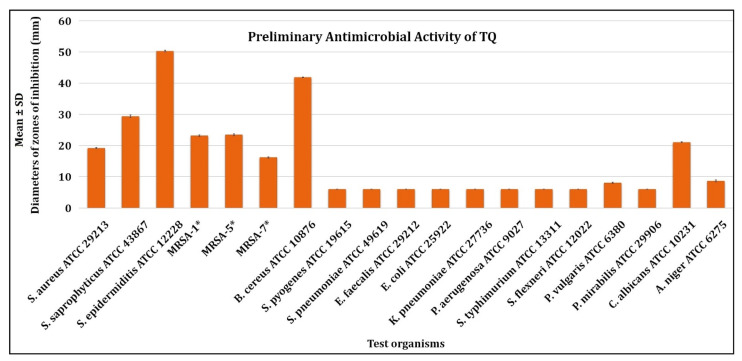
Preliminary antimicrobial activity of TQ against selected human pathogens. * MRSA-1,5,7 = *Methicillin-resistant Staphylococcus aureus* (MRSA) strains.

Preliminary antimicrobial activity tests revealed that TQ exhibits substantial antibacterial activity against all Gram-positive bacteria tested at a concentration of 200 µg/disc. This included *Staphylococcus aureus* (*S. aureus*) ATCC 29213, *Staphylococcus saprophyticus* (*S. saptophyticus*) ATCC 43867, *Staphylococcus epidermidis* (*S. epidermidis*) ATCC 12228, *Bacillus cereus* (*B. cereus*) ATCC 10876, and 03 MRSA except *Streptococcus pyogenes* (*S. pyogenes*)-A ATCC 27736, *Streptococcus pneumoniae* (*S. pneumoniae*) ATCC 49619 and *Enterococcus faecalis* (*E. faecalis*). TQ did not inhibit Gram-negative bacterial growth, with the exception of *Proteus vulgaris* (*P. vulgaris*) ATCC 6380 (Figure 2, Figure 3 and Figure S3, and Table 1). TQ also had substantial antifungal activity against *Candida albicans* (*C. albicans*) ATCC 10231 but was less effective against *Aspergillus niger* (*A. niger*) ATCC 6275 at 200 µg/disc concentration (Figure 2 and Figure 3 and Table 1). 

*Staphylococcus epidermidis* (*S. epidermidis*) ATCC 12228 was the most susceptible test bacterium tested, with a mean zone of inhibition of 50.3 ± 0.3 mm (mean ± standard deviation (SD)). At the same time, *P. vulgaris* ATCC 6380 was the least susceptible test bacterium, with a mean zone of inhibition of 8.1 ± 0.2 mm (mean ± SD) (Figure 2). *C. albicans* ATCC 10231 had a mean zone of inhibition of 21.1 ± 0.1 mm (mean ± SD), and *A. niger* ATCC 6275 a mean zone of inhibition of 8.7 ± 0.3 mm (mean ± SD). 

### 2.3. Minimum Inhibitory Concentration (MIC) and Minimum Biocidal Concentration (MBC) 

MIC and MBC results indicate that TQ has MIC values of 12.5–50 µg/mL, while the MBC values are in the range of 25–100 µg/mL, against the tested organisms (Table 2 and Figure S4). MIC data show that fungi are more susceptible to TQ than the tested bacteria. However, MBC results demonstrate that MRSA-1 and MRSA-7 need a higher dose to kill entirely than the other tested organisms. 

### 2.4. Time-Kill Kinetics Assay 

The time-kill kinetics assay revealed the antimicrobial potential of TQ and susceptibility of tested organisms toward MBC of TQ. 

**Figure 4:**
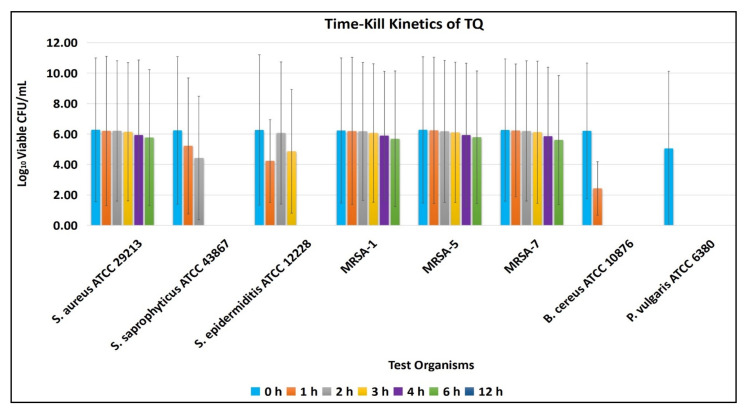
Time-kill kinetics of TQ against selected test bacteria. Note: Each test was performed in triplicate. The results are expressed in mean ± SD (log_10_ CFU/mL).

Figure 4 indicates that none of the test organisms survived until the 12th hour of incubation. The results further show that *P. vulgaris* ATCC 6380 is the most susceptible bacterium, as it could not survive until the first hour of incubation, while *S. aureus* ATCC 29213 and MRSA survived till the 6th hour of incubation with MBC of TQ. 

In conclusion, MBC and time-kill kinetics assay results suggest that TQ is bactericidal. These results are significant in deciding the dose of TQ and understanding the time duration required to kill or inhibit the tested organism’s growth at the site of infection. 

### 2.5. Minimum Biofilm Inhibitory Concentration (MBIC) and Minimum Biofilm Eradication 

#### Concentration (MBEC) 

TQ MBIC values are in the range of 25–50 µg/mL for the tested bacteria, whereas MBEC values are in the range of 25–100 µg/mL (Table 3). TQ, therefore, has a high potential to inhibit biofilm formation. These results suggest that TQ can be applied as an anti-biofilm compound in the pharmaceutical or medical industries to safeguard medical devices.

### 2.6. Statistical Analysis 

There is a statistically significant difference (*p* < 0.05) between the antimicrobial activity of TQ among the tested organisms as determined by one-way ANOVA; F (18, 38) = 16,524.945, *p* = 0.000 (Table 4).

### 2.7. In Silico Molecular Docking of TQ with Antimicrobial Enzymes

A total of 30 reported bacterial and fungal druggable target proteins were selected for MD studies with TQ [13,14,15] (Table 5). These enzymes are involved in various molecular functions, including cell wall synthesis, protein synthesis, nucleic acid synthesis, and metabolite synthesis. The inverse molecular docking studies of TQ with these potential macromolecular targets indicate that TQ is a poor binder for nitroreductase family protein from *Bacillus cereus* ATCC 14579 and spore coat polysaccharide biosynthesis protein SPSA from *Bacillus subtilis* with binding energy values greater than −5.0 kcal/mol. On the other hand, TQ binds well with bacterial D-alanyl-D-alanine synthetase (Ddl) from *Thermus thermophilus* and Transcriptional regulator qacR from *Staphylococcus aureus* as well as fungal N-myristoyltransferase from *Candida albicans* and NADPH-dependent D-xylose reductase from *Candida tenuis* with binding energy values lower than −7.0 kcal/mol, indicating its higher affinity with these proteins. The rest of the bacterial and fungal proteins exhibit moderate binding with TQ; their energies range from −5.0 to −7.0 kcal/mol. Of note, one of the bacterial proteins, isoleucyl-tRNA synthetase (IleRS) from *S. aureus,* and one of the fungal proteins, geranylgeranyltransferase type-1 subunit alpha from *Candida albicans,* exhibits lower binding energies, i.e., −7.3 and −7.6 kcal/mol, respectively. However, their lowest energy conformations do not bind into the reported protein’s binding sites, as defined by the co-crystal ligands. Therefore, TQ is less likely to inhibit these proteins during competitive inhibition. However, there are chances that TQ may function as an allosteric inhibitor, which needs further validation and experimentation.

**Figure 5:**
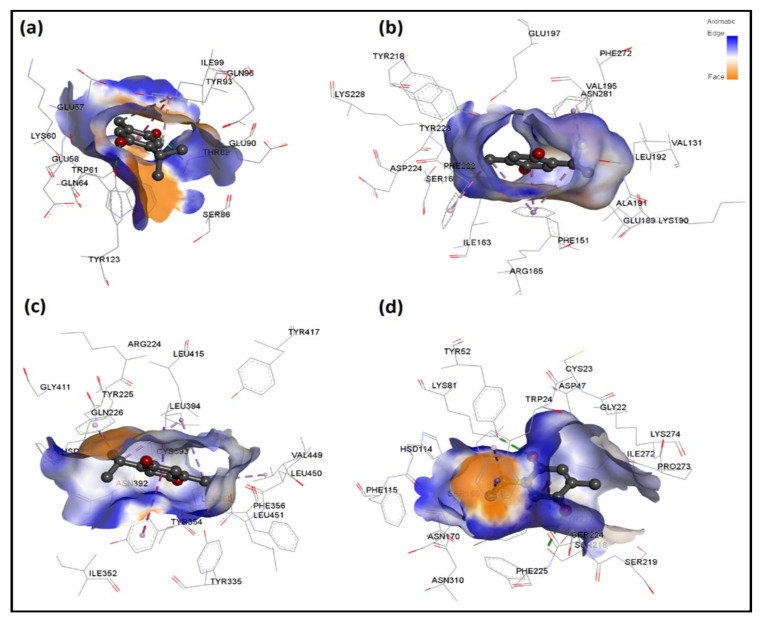
3D interaction analysis for four top-ranked TQ-protein docked systems, namely bacterial (**a**) Ddl-TQ and (**b**) qacR-TQ as well as fungal (**c**) N-myristoyltransferase-TQ and (**d**) NADPH-dependent D-xylose reductase-TQ complexes. Hydrogen bond interactions and interactions involving aromatic groups are shown in green and purple color, respectively.

Table 6 provides detailed intermolecular interactions for the four top-ranked TQ-protein docked systems. These are: bacterial Ddl-TQ or qacR-TQ and fungal N-myristoyltransferase-TQ or NADPH-dependent D-xylose reductase-TQ. Their 3D interaction images are displayed in Figure 5. It is clear that TQ predominantly forms hydrophobic interactions with qacR and N-myristoyltransferase, while with Ddl and NADPH-dependent D-xylose reductase, hydrogen bonds are also formed. All four top-ranked complexes were subjected to MD simulations to further validate the ligand’s binding and evaluate its binding strength to target proteins.

#### 2.7.1. MD Simulations of TQ-Enzyme Complexes

Four separate MD simulations were carried out for 100 ns each to understand the behavior of TQ-induced effects and its binding with respective target proteins. The trajectories were analyzed in terms of root mean square deviations (RMSD), root mean square fluctuations (RMSF), TQ-protein center-of-masses (CoM) distance, radius of gyration (g_(r)_), solvent accessible surface area (SASA), TQ-protein hydrogen bond formation, and molecular mechanics/generalized-born surface area (MM/GBSA) binding energy.

##### Protein and TQ Stability

**Figure 6:**
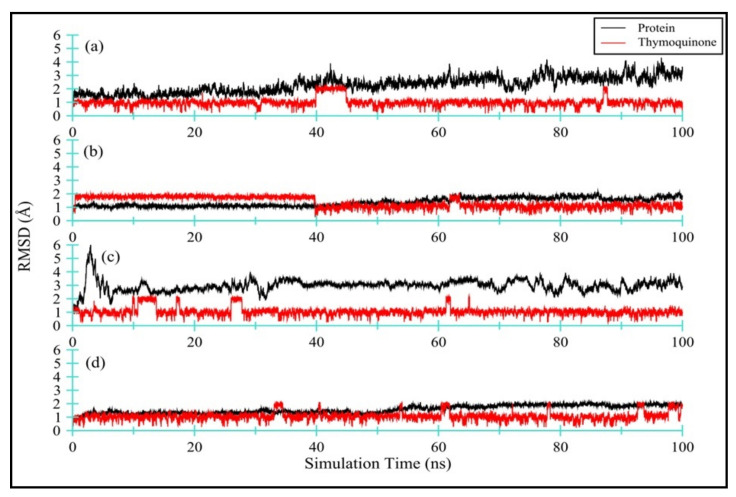
RMSD for all four TQ-enzyme complexes, namely bacterial (**a**) DdI-TQ and (**b**) qacR-TQ as well as fungal (**c**) N-myristoyltransferase-TQ and (**d**) NADPH-dependent D-xylose reductase-TQ. The RMSD for enzymes is black in color, while TQ is red in color.

Figure 6 displays the RMSD for all four TQ-enzyme complexes, namely (a) Ddl-TQ, (b) qacR-TQ, (c) N-myristoyltransferase-TQ, and (d) NADPH-dependent D-xylose reductase-TQ. During 100 ns of simulation, the RMSD plot for Ddl (a; black line) shows a slight increase until 40 ns before plateauing. The mean RMSD value was observed as 2.30 ± 0.59 Å. On the other hand, the RMSD plot for qacR (b; black line) shows a stable pattern with a mean RMSD value of 1.37 ± 0.30 Å. The N-myristoyltransferase protein has a long unstructured N-terminus, manifested in strong deviation within the first 7 ns of simulation time (c; black line). After 7 ns until 100 ns of simulation time, the RMSD plot remains stable with few noticeable fluctuations. The mean RMSD value was calculated as 2.97 ± 0.44 Å. Unlike N-myristoyltransferase, the overall RMSD plot for NADPH-dependent D-xylose reductase (d; black line) shows a stable curve with the mean RMSD value as 1.56 ± 0.30 Å. Overall, all proteins exhibit small deviation values (1.56 ≥ RMSD_(av)_ ≤ 2.30 Å) from the initial structure, except for the N-myristoyltransferase-TQ complex (c; black line), where the RMSD value is slightly higher (RMSD_(av)_ = 2.97 Å) due to an extremely flexible long N-terminus. 

Figure S4 illustrates a comparison of all four proteins, namely bacterial (a) Ddl and (b) qacR as well as fungal (c) N-myristoyltransferase and (d) NADPH-dependent D-xylose reductase at 0 ns (initial conformation; opaque representation) and 100 ns (final conformation; transparent representation) of simulation time. This indicates how the proteins have deviated from the starting conformations.

Figure 6 also displays (in red) the RMSD for TQ ligand complexed with: (a) Ddl-TQ, (b) qacR-TQ, (c) N-myristoyltransferase-TQ, and (d) NADPH-dependent D-xylose reductase-TQ. The mean RMSD values ranged from 1.0 to 1.34 Å for all four ligands. Among all four complexes, TQ in complex with qacR protein (b; red line) displays a slightly higher RMSD and standard deviation values (i.e., 1.34 ± 0.40 Å), indicating that the TQ is occupying various conformational states and positions within the binding site. Figure S5 clearly depicts the formation of two clusters of TQ in the binding site. The TQ in the other three complexes shows relatively lower RMSD and standard deviation values, and they predominantly form one cluster within the binding site (Figure S5).

##### TQ-Induced Protein Flexibility

**Figure 7:**
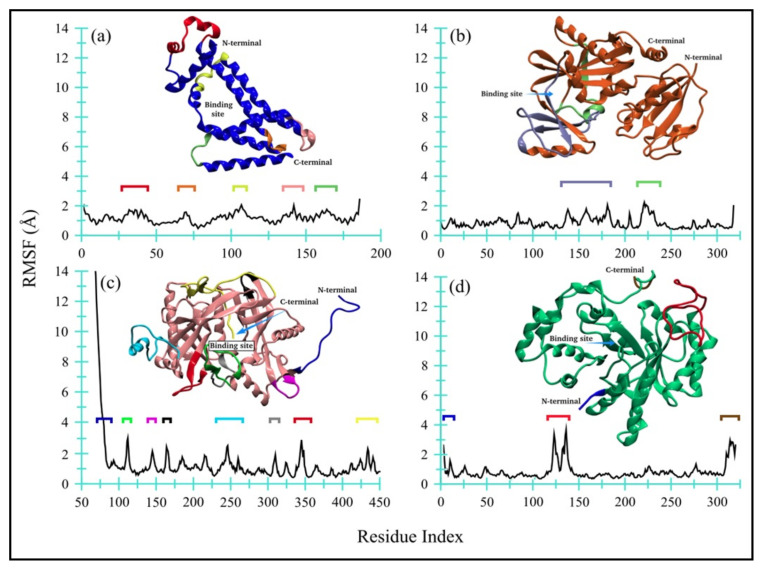
Root mean square fluctuations for all four enzyme complexes: (**a**) Ddl, (**b**) qacR, (**c**) N-myristoyltransferase, and (**d**) NADPH-dependent D-xylose reductase.

Figure 7 displays the RMSF for all four enzyme complexes, namely bacterial (a) Ddl and (b) qacR as well as fungal (c) N-myristoyltransferase and (d) NADPH-dependent D-xylose reductase. The flexible regions have also been highlighted in different colors. Overall, both bacterial proteins, i.e., (a) D-alanyl-D-alanine synthetase and (b) qacR, remained quite contained and only fluctuated within the range of 0.4 to 2.4 Å (Δ = 2 Å), with mean RMSF values of 1.19 ± 0.32 Å and 0.84 ± 0.36 Å, respectively. A modest ligand-induced conformational flexibility can also be seen in qacR (b), where residues near the binding site regions (colored purple and green) exhibit greater RMSF values as compared to the other regions of the protein.

On the other hand, one of the fungal proteins, i.e., (c) N-myristoyltransferase, exhibited comparatively large RMSF values for loop, short-helices, and part of beta-strands, indicating a flexible structure of the protein during MD simulation. The unstructured long-chain N-terminus showed greater flexibility, with the RMSF value reaching up to ~14 Å. The ligand-induced effects are also moderately prominent in residues near the binding site (colored green). The mean RMSF value, however, remained at 1.29 ± 1.53 Å. Contrary to this, the NADPH-dependent D-xylose reductase (d) remained quite contained, except for a large loop region (colored red), like bacterial proteins with a mean RMSF value of 0.83 ± 0.55 Å. Both N- and C-terminals are moderately flexible. Overall, no global changes are observed in protein, while the ligand-induced conformational changes are noticeable for qacR-TQ (b) and N-myristoyltransferase-TQ (c) complexes. 

##### Distance Fluctuation between TQ and Enzymes and TQ Dynamics

**Figure 8:**
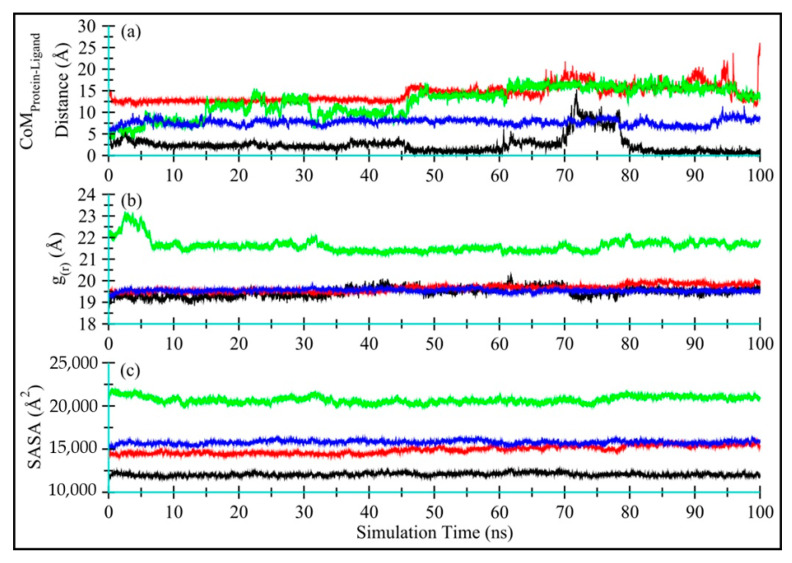
Time-dependent analyses for Ddl-TQ (colored black), qacR-TQ (colored red), N-myristoyltransferase-TQ (colored green), and NADPH-dependent D-xylose reductase-TQ (colored blue) complexes: (**a**) The distance between the center-of-masses of TQ and enzyme during 100 ns of MD simulation; (**b**) The radius of gyration; and (**c**) Solvent-accessible surface area for proteins in complex with TQ.

The distance between the CoM of TQ and enzyme, as displayed in Figure 8a, indicates that TQ remains bound with all enzymes, with average CoM distances of 2.51 ± 2.02 Å, 14.34 ± 1.92 Å, 12.62 ± 3.33 Å, and 7.71 ± 0.73 Å, for Ddl (black), qacR (red), N-myristoyltransferase (green) and NADPH-dependent D-xylose reductase (blue), respectively. The higher standard deviations show that the ligand adopts various conformations and occupies multiple positions within the binding space of the protein center. Figure 8a–d shows ligand conformation within the proteins binding site. 

##### Global Conformational Changes in Enzymes

Figure 8b displays the radius of gyration (g_(r)_) for Ddl-TQ (colored black), qacR-TQ (colored red), N-myristoyltransferase-TQ (colored green), and NADPH-dependent D-xylose reductase-TQ (colored blue) complexes. No significant global change was observed in terms of protein compactness in all four proteins during 100 ns MD simulations. N-myristoyltransferase-TQ (colored green) did show slight fluctuations only in the first 7 ns of simulation time, indicating noticeable changes in protein compactness due to the long unstructured N-terminus, further endorsing the RMSD and RMSF plots. The mean g_(r)_ values were observed as 19.47 ± 0.21 Å, 19.65 ± 0.16 Å, 21.62 ± 0.30 Å, and 19.55 ± 0.07 Å, respectively for Ddl-TQ, qacR-TQ, N-myristoyltransferase-TQ, and NADPH-dependent D-xylose reductase-TQ. 

Figure 8c displays the solvent-accessible surface (SASA) for all four proteins (Ddl-TQ, qacR-TQ, N-myristoyltransferase-TQ, and NADPH-dependent D-xylose reductase-TQ). Similarly to g_(r),_ no significant change in SASA for all four proteins was observed, indicating that no significant part of the proteins was exposed to water, and the structure remained compact throughout the simulation time. The mean SASA values for Ddl-TQ, qacR-TQ, N-myristoyltransferase-TQ, and NADPH-dependent D-xylose reductase-TQ were: 12,066 ± 181 Å, 14,914 ± 443 Å, 20,721 ± 377 Å and 15,785 ± 212 Å, respectively.

##### Enzyme-TQ Binding Energy

**Figure 9:**
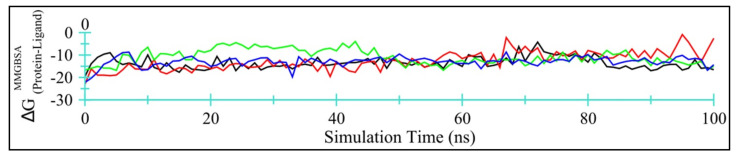
Time-dependent MM/GBSA binding energy (kcal/mol) for Ddl-TQ (colored black), qacR-TQ (colored red), N-myristoyltransferase-TQ (colored green), and NADPH-dependent D-xylose reductase-TQ (colored blue) complexes.

Figure 9 displays time-dependent MM/GBSA plots for Ddl-TQ (colored black), qacR-TQ (colored red), N-myristoyltransferase-TQ (colored green), and NADPH-dependent D-xylose reductase-TQ (colored blue) complexes. 

Table 7 summarizes the energy contributions form van der Waal’s, electrostatics, polar, and non-polar solvation free energies. Evidently, van der Waal’s interactions account for the majority of the binding energy. Among all four target proteins, TQ binds more efficiently with Ddl and NADPH-dependent D-xylose reductase, with lower MM/GBSA values and smaller standard deviations. Overall, TQ may be regarded as a moderate inhibitor for these four proteins. 

## 3. Discussion

MDR microorganisms have become a significant public health concern due to their rapid spread over the past decade. Antimicrobial resistance develops quickly in microorganisms, and most synthetic drugs have adverse effects on the human body [8,12]. Due to the emergence of multi-drug resistance and the lack of new safe antimicrobials, innovative strategies for treating MDR pathogens are needed, with low side effects. 

TQ is a core bioactive phytoconstituent of *Nigella sativa* essential oil, and it has been reported that it has substantial antimicrobial potential against various human pathogens. TQ has several pharmacological applications, including anti-inflammatory, anticancer, antidiabetic, anti-asthmatic, hypolipidemic, anti-hypertensive, and nephroprotective properties [11].

The antimicrobial evaluation of TQ was carried out with the resazurin-based 96-well plates. This technique is commonly used to determine the MIC, time-kill assay, MBIC, and MBEC and has some limitations, including the fact that it is an indirect method for determining the viability of cells in the well; consequently, the results must be verified by viable cell counting from the contents of the analyzed well or by additional waiting for 24 h at 35 °C to observe the well’s color change from blue to pink, indicating the presence of metabolically active cells. In our case, we chose additional waiting for 24 h and found no color change from blue to pink; thus, results were unchanged. As a result, our findings are also valid. However, we strongly recommend that results be validated using CFU (colony forming unit) counts.

The in vitro antimicrobial activity of TQ is supported by in silico molecular docking and MD simulation studies. However, the in silico studies could provide insights into the molecular mechanism of antimicrobial activity. The in silico analyses have some limitations, and thus, all the computational analyses must be validated by some additional in vitro experimental studies, e.g., X-ray crystallography, etc. 

This study shows that TQ has substantial antimicrobial and anti-biofilm potential against various selected human pathogens. Time-kill assay reveals the time required to kill susceptible pathogens in the optimal experimental conditions, which can be helpful during the management of various infections through the applications of TQ and TQ-based therapeutics. 

Our findings are consistent with previously published studies [8,12,36]. According to Halawani (2009), TQ exerts antibacterial activity against Gram-positive bacteria. The author demonstrated that *S. aureus* is highly susceptible to TQ with MIC and MBC of 3 and 6 µg/mL, and Gram-negative bacteria were less susceptible to TQ with MIC and MBC ranging from 200 to 1600 µg/mL. These findings corroborate our findings that TQ has significant antibacterial activity against Gram-positive bacteria and that Gram-negative bacteria are resistant to TQ [8]. Chaieb et al. (2011) demonstrated that TQ exhibits substantial bactericidal activity against *S. aureus* ATCC 25923 and *S. epidermidis* CIP 106510 with MIC values ranging from 8 to 32 μg/mL. Additionally, they found that TQ has MBIC_50_ values of 22 and 60 μg/mL for *S. aureus* ATCC 25923 and *S. epidermidis* CIP 106510, respectively. These findings corroborate our findings that the MIC for *S. aureus* ATCC 29213 and *S. epidermidis* ATCC 12228 is 50 μg/mL, and the MBIC is also the same as 50 μg/mL [36]. Dera et al. (2021) found that TQ is effective against *K. pneumoniae*, *S. epidermidis* ATCC 12228, *S. aureus*, and *S. epidermidis* with MIC ranging from 1.04 to 8.3 μg/mL and MBC ranging from 10.41 to 66.66 μg/mL. They also showed that TQ inhibited the formation of biofilms after treatment with various TQ concentrations against the tested bacterial strains. Additionally, they found that TQ is not effective against *Enterococcus faecalis* ATCC 29212, *M. smegmatis*, *S. saprophyticus*, *S. pyogenes*, *E. coli* ATCC 25922, *P. aeruginosa* ATCC 27853, *E. coli*, *Pseudomonas* sp., *Salmonella typhi*, and *Shigella* sp. at a given concentration of 50 μg/mL [12]. These results are consistent with our results demonstrating that TQ is not substantially effective against Gram-negative bacteria, *S. pyogenes,* and *Enterococcus faecalis*. In contrast, our results showed that TQ is effective against *S. saprophyticus*, whereas they reported *S. saprophyticus* as resistant to TQ. 

## 4. Materials and Methods

### 4.1. Chemicals and Reagents 

TQ was procured from Sigma-Aldrich (Milwaukee, Wis., USA) and had a purity of 99.0%. FT-IR was performed to validate the identity and purity of TQ [1]. The penicillin (2 µg), erythromycin (10 µg), ampicillin (30 µg), amoxicillin (25 µg), amoxycillin/clavulanic acid (30 µg), cefotaxime (30 µg), gentamycin (10 µg), chloramphenicol (30 µg), tetracycline (30 µg), and imipenem (10 µg) antibiotic discs were procured from Oxoid Limited, United Kingdom. 

### 4.2. Test Organisms

All 16 ATCC (American Type Culture Collection) test organisms were procured from Microbiologics, Biotechnology Company (200 Cooper Ave N, St Cloud, MN 56303, USA), while 03 clinical isolates (MRSA) were received from the microbiology department of King Saud Hospital, Unaizah, Saudi Arabia. *S. aureus* ATCC 29213, *S. saprophyticus* ATCC 43867, *S. epidermidis* ATCC 12228, MRSA-1, MRSA-5, MRSA*-7, B. cereus* ATCC 10876, *S. pyogenes*-A ATCC 19615, *S. pneumoniae* ATCC 49619, *E. faecalis* ATCC 29212, *E. coli* ATCC 25922, *K. pneumoniae* ATCC 27736, *P. aerugenosa* ATCC 9027, *S. typhimurium* ATCC 13311, *S. flexneri* ATCC 12022, *P. vulgaris* ATCC 6380, *P. mirabilis* ATCC 29906, *C. albicans* ATCC 10231, and *A. niger* ATCC 6275 were used as test organisms. Additionally, the antimicrobial susceptibility pattern of all the tested bacterial strains was determined by the disc-diffusion method with some standard antibiotics [1]. 

### 4.3. Preliminary Antimicrobial Activity 

The preliminary antimicrobial activity of TQ was determined by the disc diffusion method [1,4,37,38,39]. Mueller–Hinton agar (MMHA) and potato dextrose agar (PDA) were used as test media. MMHA was prepared by dissolving 19.0 g of dehydrated Mueller–Hinton agar (MHA) base and 18.0 g of CLED (cystine–lactose–electrolyte–deficient) agar base in 1 L of ultrapure deionized water, and the pH was adjusted to 7.0 ± 0.2. MMHA is a highly supportive medium for the growth of fastidious organisms and offers enough contrast for image acquisition. A stock solution of TQ was prepared in DMSO with a 10 mg/mL concentration. Twenty microliters of the diluted TQ solution was then dispensed on each tested sterile disc. Thus, each disc consisted of 200 µg of TQ, while the control discs (C) were prepared by dispensing 20 µL of DMSO/disc. Each organism’s inoculum was prepared in sterile tryptic soy broth (TSB), and the turbidity of each suspension was adjusted equal to 0.5 MacFarland standard at OD_600_ (0.08–0.12). Then, 100 µL each of the adjusted inoculum was dispensed onto an MMHA plate, separately, and then suspensions were evenly distributed using sterile swabs. After that, the prepared discs of TQ and C were placed on the surface of inoculated plates. All the plates were incubated at 35 °C for 24 h for bacteria and 48 h for fungi. After incubation, the diameters of inhibitory zones were measured on a millimeter (mm) scale. Each test was performed in triplicate. The results were expressed in mm ± SD. 

### 4.4. MIC and MBC 

MIC was determined by the resazurin-based micro broth dilution method, while MBC was performed following the standard spot inoculation method [1,2,4,40,41]. The inocula of each test bacteria were prepared in TSB, following the CLSI guidelines, where the OD_600_ value (0.08–0.12) was adjusted, resulting in ~1 × 10^8^ CFU/mL. Then adjusted inocula were further diluted by 1:100 in TSB, resulting in ~1 × 10^6^ CFU/mL. In contrast, the inocula of test fungi were prepared in potato dextrose broth (PDB) following the CLSI guidelines, where the OD_600_ value (0.08–0.12) was adjusted, the resulting stock suspension contained 1 × 10^6^ to 5 × 10^6^ CFU/mL for yeast and 4 × 10^5^ to 5 × 10^6^ CFU/mL for mold. A working yeast suspension was prepared by a 1:100 dilution followed by a 1:20 dilution of the stock suspension with PDB, resulting in 5.0 × 10^2^ to 2.5 × 10^3^ cells/ mL, while a working mold suspension was prepared by a 1:50 dilution of the stock suspension with PDB, resulting in 0.8 × 10^4^ to 1 × 10^5^ cells/ mL. The stock solution of TQ was prepared in DMSO with a 200 µg/mL concentration, and then 200 µL stock solution was dispensed in each well of column 1, while columns 2–10 contained 100 µL of TSB only. Column 11 had 200 µL of standardized inoculum suspensions, which served as negative control (NC), and column 12 had 200 µL of sterile broth, which served as sterility control (SC). A twofold serial dilution was prepared by mixing and transferring the TQ solution from column 1 to 10 with a multichannel pipette, yielding 100 µL/well. The tested concentrations of the TQ achieved through a twofold serial dilution from columns 1–10 were 100–0.049 µg/mL. The 100 µL of adjusted microbial inocula were dispensed in all the wells of columns 1–10, resulting in ~ 5 × 10^5^ CFU/mL for bacteria and ~2.5 × 10^2^ to 1.25 × 10^3^ CFU/mL for *C. albicans*, and 0.4 × 10^4^ to 5 × 10^4^ CFU/mL for *A. niger*. The time taken to prepare and dispense the OD-adjusted microbial inocula did not exceed 15 min. All inoculated plates were incubated at 35 °C for 24 h for bacteria and 48 h for fungi. Following the incubation, the 30 µL of sterile resazurin (0.015%, *w*/*v*) solution was dispensed in each well and again incubated for 1–2 h to observe color change. Following incubation, the columns that remained blue in color were recorded as MIC. MBC was determined by directly plating the contents of wells with concentrations above the MIC on sterile tryptic soy agar (TSA) plates for bacteria, while potato dextrose agar (PDA) plates for fungi. The lowest concentration of TQ did not produce isolated colonies of the test organisms on inoculated agar plates considered the MBC.

### 4.5. Time-Kill Kinetics Assay

A modified time-kill kinetics method was used to determine the time-kill kinetics values of TQ against the tested bacteria [8,42,43,44,45]. Using this method, we selected MBC values of TQ for each tested bacteria and followed the same protocol as we did when evaluating MIC. Bacterial growth was quantified after 0, 1, 2, 3, 4, 6, and 12 h of incubation at 37 °C by plating 10-fold dilutions on TSA. Each test was performed in triplicate. The results are expressed in log_10_ viable CFU/mL. 

### 4.6. MBIC and MBEC Assay of TQ

MBIC is defined as the lowest concentration of the antimicrobial agent, preventing the biofilm formation of the tested organism. MBIC was conducted against the bacteria only. The 96-well microtiter plate was used to evaluate the anti-biofilm activity of TQ [45]. The inocula of the test organisms were prepared in TSB equal to 0.5 MacFarland standard (1–2 × 10^8^ CFU/mL). An aliquot of 100 μL from the adjusted inocula was dispensed into each test well of a 96-well plate. Then 100 μL of different concentrations of TQ were dispensed into test wells. Thus, the final concentrations for MBIC assessment were MIC, 2 × MIC, and 4 × MIC. The wells containing only 200 μL of TSB served as a blank control (BC), whereas those containing bacterial cultures without TQ served as negative control (NC). The plates were incubated in a shaking water bath at 35 °C for 24 h at 100 rpm shaking speed. After incubation, the supernatants from each well were decanted gently by reversing the plates on a tissue paper bed/or removed by a pipette without disturbing the biofilms. The plates were dried in air for 30 min, stained with 0.1% (*w*/*v*) crystal violet at room temperature for 30 min, and then washed three times with distilled water. Subsequently, the crystal violet was solubilized by adding 200 µL of 95% ethanol in each test well. The absorbance was recorded in a microplate reader (xMark™ Microplate Absorbance Spectrophotometer-Bio-Rad, Hercules, CA, USA) at 650 nm. The lowest concentration of TQ at which the absorbance equals or falls below that of the negative control is considered MBIC. Each test was performed in triplicate. The mean of three independent tests was taken. The results are expressed in µg/mL. 

MBEC is defined as the minimum concentration of an antimicrobial agent that eradicates the biofilm of the test organism [45]. A 200 μL (1–2 × 10^8^ CFU/mL) inoculum of each test organism was inoculated into each test well of a flat-bottom 96-well microtiter plate. The plates were incubated at 35 °C for 48 h in a shaking water bath at 100 rpm shaking speed for biofilm formation. After forming the biofilms, the contents of the test wells were decanted gently by reversing the plates on a tissue paper bed/or removed by a pipette without disturbing the biofilms. The various concentrations, i.e., MIC, 2 × MIC, and 4 × MIC of TQ, were added to different test wells (200 μL/well). The inoculated plates were re-incubated at 35 °C for 24 h. After incubation, the contents of each test wells were discarded by inverting the plates on a tissue bed. The plates were dried in air for 30 min, and then 200 μL of sterile TSB was dispensed in each test well. Then 30 µL of 0.015% *w*/*v* resazurin dye was added into each test well. The plates were re-incubated for 1–2 h. After re-incubation, the MBEC values were recorded by observing the color change from blue to pink. The column with no color change (blue resazurin color stayed intact) was scored MBEC. Biofilm without TQ served as a positive control. Each test was performed in triplicate. The mean of three independent tests was taken. The results are expressed in µg/mL. 

### 4.7. Statistical Analysis

The preliminary antimicrobial activity of TQ was statistically analyzed using the one-way ANOVA statistical test to determine statistical differences among the means of tested organisms. The post hoc test (Tukey’s method) was performed to assess the significance of interactions among the means of groups, where *p* = 0.05 was considered statistically significant. The SPSS software, version 20.0 (IBM Corp., Armonk, NY, USA), was used to conduct the statistical analysis.

### 4.8. In Silico Molecular Dockings of TQ 

#### 4.8.1. Identification of Target Proteins

An open web server PharmMapper was used to identify possible TQ targets through reverse pharmacophore mapping [13,14,15]. PharmMapper is supported by a huge, in-house repertoire of the pharmacophore database derived from all the targets in TargetBank, DrugBank, BindingDB, and PDTD with 16,159 druggable and 52,431 ligandable pharmacophore models. PharmMapper identifies the optimal mapping poses of the user-submitted molecules in Tripos/Mol2 or MDL/SDF format against all the targets in PharmTargetDB and the top N possible drug targets corresponding to the molecule’s aligned poses are generated. It provides results with a Z score according to the similarity of pharmacophore to the query compound, with the identified target pharmacophore model and the importance of target proteins in diseases [13,14,15]. 

TQ was submitted to PharmMapper to identify its possible drug targets. The selection of targets was based strictly on their association with bacteria and fungi. The target proteins retrieved were ranked according to their fitness score. The top 30 proteins with a fitness score of more than 2.0 were studied to identify possible TQ targets. The 3D structure of TQ was downloaded from PubChem (CID 10281) [46]. The Gasteiger–Marsili empirical atomic partial charges (determined based on electronegativity equilibration) [47] were defined for ligand, and the .pdbqt file was generated using Raccoon [48]. 

Twenty bacterial and 10 fungal reported druggable proteins were downloaded from the protein data bank (PDB); their PDB IDs are given in Table 5. Proteins with missing residues were first searched in the AlphaFold protein structure database [49], and those which were unavailable were subjected to loop construction using self-template-based homology modeling using SWISS-MODEL [50]. The water, ions, and other impurities were removed from the protein files, and relevant chain/s were extracted.

Where applicable, the coordinates of co-crystal ligands were recognized as protein binding site regions, and the search space was defined around it. In the case that no co-crystal ligand was present, a blind docking was performed, and the search space was extended to cover the whole protein molecule. Autodock tools were used to generate search space box and .pdbqt files by applying Merz–Singh–Kollman partial charges (derived from the corresponding molecular electrostatic potential, MEP, using quantum mechanics) [51] for protein molecules. During protein file preparation, the grid point spacing was increased from 0.375 to 1.0 in AutoDock Tools [52]. The Autodock vina [53] was used for molecular docking, and the value for exhaustiveness was increased to 80 to decrease the probability of not finding the global minimum. A total of 20 docked conformations was recorded for each complex system for further analysis. The top four best complexes (two from each bacterial and fungal protein) with the lowest binding energies were subjected to a MD simulation study. Table 5 lists the binding energy of all docked complexes.

### 4.9. MD Simulations Protocol

The top-ranked TQ-protein complexes, namely Ddl, qacR, N-myristoyltransferase, and NADPH-dependent D-xylose reductase, were subjected to MD simulation. All four protein–TQ complex systems were immersed in the truncated octahedral box containing TIP3P water molecules. The minimum distance between protein systems and the edges of the simulation box was set to 10 Å to efficiently meet the criteria for minimum image convention during MD simulation. All four protein complex systems were electronically neutralized by adding 8 K^+^ ions in the environment. We ensured that the Cysteine–Cysteine disulfide bonds between Cys298-Cys310, Cys301-Cys306, and Cys487-Cys645 had been created, using the webserver tool of Beijing Computational Science Research Center, China, followed by the manual validation. The algorithm for predicting disulfide bonds in protein molecules is based on machine learning image classification methods, which utilize statistical information from the existing PDB structures [16]. The protonation states were evaluated for His, Lys, Arg, Asp, and Glu residues at 7.4 pH using https://playmolecule.com/ (accessed on 12 September 2021) [54] protein prepared web server and implemented after visual inspection. The bacterial complexes (Ddl-TQ and qacR-TQ) and fungal complexes (N-myristoyltransferase-TQ and NADPH-dependent D-xylose reductase-TQ) contained 195,702, 195,648, and 195,691 atoms in total, respectively. The Chemistry at HARvard Macromolecular Mechanics Graphical User Interface (CHARMM-GUI) webserver was used to generate all input files [55].

All systems were minimized for 5000 steps using the Steepest Descent technique, and convergence was achieved under the force limit of 1000 (kJ/mol/nm) to exclude any steric clashes. Later, all four minimized systems were separately equilibrated at NVT (Canonical ensemble: where moles, N; volume, V; and temperature, T were conserved) and NPT (Isothermal-Isobaric ensemble: where moles, N; pressure, P; and temperature, T were conserved) ensembles for 100 ps (50,000 steps) and 1000 ps (1,000,000 steps), respectively, using time steps 0.2 and 0.1 fs, at 300 K to ensure a fully converged systems for the production run [56].

The simulation runs for all four systems were conducted at a constant temperature of 300 K and a pressure of 1 atm, or 1 bar (using an NPT ensemble), utilizing weak coupling velocity re-scaling (modified Berendsen thermostat) and Parrinello–Rahman algorithms, respectively. The relaxation times were set at τ T = 0.1 ps and τ P = 2.0 ps. Using the LINear Constraint Solver (LINCS) algorithm, all bond lengths involving hydrogen atoms were maintained stiffly at optimal lengths, with a time step of 2 fs. The non-bonded interactions were calculated using the Verlet algorithm. Interactions within a short-range cutoff of 12 Å were calculated in each time step. The electrostatic interactions and forces in a homogeneous medium beyond the short-range cutoff were calculated using the Particle Mesh Ewald (PME) method. Periodic Boundary Conditions (PBC) were applied in all x, y, and z directions. For each of the three complexes, the production was run for 200 ns. The trajectory and energy data were recorded every 10 ps [56]. GROMACS simulation package (GROMACS 2020.4) [57,58] was used to perform MD simulations using CHARMM36m forcefield [59]. GRaphing, Advanced Computation and Exploration of data (Grace) was used to generate all plots (https://plasma-gate.weizmann.ac.il/Grace, accessed on 12 September 2021). The Molecular Mechanics/Generalized-Born Surface Area (MM/GBSA) [60] protein–ligand binding energy was calculated after every 1 ns of simulations for all four systems. The following equation was used to calculate Gibb’s binding free energy using the single trajectory method described by Genheden and Ryde [60].

∆*G*_*binding*_ = *G*_*complex*_ − *G*_*protein*_ − *G*_*ligand*_

where *G* can be calculated as:

*G* = *E*_*ele*_ + *E*_*vdW*_ + *G*_*pol*_ + *G*_*np*_ − *TS*

where the *E*_*ele*_ and *E*_*vdW*_ are standard MM energy terms representing electrostatic and Van der Waal’s interactions. Since the bonded terms are canceled out in the single trajectory approach, they are not mentioned here. *G*_pol_ and *G*_*np*_ are the polar and non-polar contributions to the solvation free energies, calculated using the Generalized-Born model and obtained from a linear relation to the SASA, respectively. *TS* is the configurational entropy, which measures the number of available configurations occupied by a molecule in 3D space, multiplied by the temperature, and it is typically ignored because of the associated higher computational cost. This method of Gibb’s free energy calculation is widely accepted and used in colossal studies.

## 5. Conclusions

In conclusion, the results of this study indicate that TQ has substantial antimicrobial potential and can be used to treat various bacterial infections caused by Gram-positive bacteria, including MRSA and fungal infections caused by *Candida albicans*. This study further shows that TQ can be utilized as an anti-biofilm agent to inhibit biofilm formation on various medical devices, including catheter tips and dental implants. As a result of our findings, we suggest that additional exploration of TQ for usage in the clinic is warranted.

## Figures and Tables

**Table 1:** Preliminary antimicrobial potential of TQ against selected human pathogens.

Microorganism	Zone of Inhibition Diameter (mm)	
	TQ (200 µg/disc)	Control (20 µL/disc)
*S. aureus* ATCC 29213	19.2 ± 0.2	6.0 ± 0.0
*S. saprophyticus* ATCC 43867	29.4 ± 0.4	6.5 ± 0.1
*S. epidermidis* ATCC 12228	50.3 ± 0.3	7.0 ± 0.2
MRSA-1 *	23.2 ± 0.2	6.0 ± 0.0
MRSA-5 *	23.5 ± 0.3	7.0 ± 0.1
MRSA-7 *	16.2 ± 0.2	6.0 ± 0.0
*B. cereus* ATCC 10876	41.9 ± 0.1	6.5 ± 0.2
*S. pyogenes*-A ATCC 19615	6.0 ± 0.0	6.0 ± 0.0
*S. pneumoniae* ATCC 49619	6.0 ± 0.0	6.0 ± 0.0
*E. faecalis* ATCC 29212	6.0 ± 0.0	6.5 ± 0.2
*E. coli* ATCC 25922	6.0 ± 0.0	6.0 ± 0.0
*K. pneumoniae* ATCC 27736	6.0 ± 0.0	6.5 ± 0.1
*P. aerugenosa* ATCC 9027	6.0 ± 0.0	6.0 ± 0.0
*S. typhimurium* ATCC 13311	6.0 ± 0.0	6.0 ± 0.0
*S. flexneri* ATCC 12022	6.0 ± 0.0	6.0 ± 0.0
*P. vulgaris* ATCC 6380	8.1 ± 0.2	6.5 ± 0.1
*P. mirabilis* ATCC 29906	6.0 ± 0.0	6.5 ± 0.2
*C. albicans* ATCC 10231	21.1 ± 0.1	6.0 ± 0.0
*A. niger* ATCC 6275	8.7 ± 0.3	6.0 ± 0.0

Note: “6.0 ± 0.0” indicates no zone of inhibition. Each test was performed in triplicate. All results are in mean ± SD. * Clinical isolates. Control = Dimethyl sulfoxide (DMSO).

**Table 2:** MIC and MBC results of TQ against selected human pathogens.

Microorganism	MIC (µg/mL)	MBC (µg/mL)
*S. aureus* ATCC 29213	50	50
*S. saprophyticus* ATCC 43867	25	50
*S. epidermidis* ATCC 12228	50	50
MRSA-1	50	100
MRSA-5	25	50
MRSA-7	50	100
*B. cereus* ATCC 10876	25	25
*P. vulgaris* ATCC 6380	25	50
*C. albicans* ATCC 10231	12.5	25
*A. niger* ATCC 6275	12.5	25

**Table 3:** MBIC and MBEC of TQ against selected test bacteria.

Microorganisms	MBIC (µg/mL)	MBEC (µg/mL)
*S. aureus* ATCC 29213	50	50
*S. saprophyticus* ATCC 43867	25	50
*S. epidermidis* ATCC 12228	50	50
MRSA-1	50	100
MRSA-5	25	50
MRSA-7	50	100
*B. cereus* ATCC 10876	25	25
*P. vulgaris* ATCC 6380	25	50
*C. albicans* ATCC 10231	NT	NT
*A. niger* ATCC 6275	NT	NT

Note: NT = Not tested.

**Table 4:** One-way ANOVA for the preliminary antimicrobial potential of TQ.

ANOVA					
	Sum of Squares	df	Mean Square	F	Sig.
Between Groups	9549.679	18	530.538	16,524.945	0.000
Within Groups	1.220	38	0.032		
Total	9550.899	56			

**Table 5:** Some structural and functional details of the selected bacterial and fungal proteins used for molecular docking studies with TQ. The binding energy of each TQ-ligand complex is also given, as calculated from the Autodock Vina molecular docking tool.

#	Target Proteins	PDB ID	Source Organism	Function	Reference	Binding Energy from Docking (kcal/mol)
**Bacterial Protein Targets**						
1	Alanine racemase (Alr)	2SFP	*Geobacillus stearothermophilus*	Cell wall synthesis	[16]	−6.5
2	D-alanyl-D-alanine synthetase (Ddl)	2ZDQ	*Thermus thermophilus*	Cell wall synthesis	[16]	−7.8
3	Penicillin-binding protein 3 (PBP3)	3VSL	*Methicillin-Resistant Staphylococcus aureus*	Cell wall synthesis	[17]	−5.2
4	Transcriptional regulator (TcaR)	3KP3	*Staphylococcus epidermidis RP62A*	Protein biosynthesis	[18]	−6.2
5	Penicillin-binding protein 1a (PBP1a)	3UDI	*Acinetobacter baumannii*	Cell wall synthesis	[16]	−6.7
6	Dihydrofolate reductase (DHFR)	3SRW	*Staphylococcus aureus*	Metabolite synthesis	[16]	−6.3
7	Dihydropteroate synthetase (DHPS)	2VEG	*Streptococcus pneumoniae*	Metabolite synthesis	[16]	−5.2
8	DNA gyrase subunit B	3TTZ	*Staphylococcus aureus*	Nucleic acid synthesis	[16]	−5.5
9	Topoisomerase IV (TopoIV)	3RAE	*Streptococcus pneumoniae*	Nucleic acid synthesis	[16]	−5.5
10	Sortase A	2MLM	*Staphylococcus aureus CA-347*	(1) Attach proteins to the cell wall and (2) join proteins together to construct pili.	[19]	−5.7
11	Glycerol phosphate lipoteichoic acid synthase 2	2W8D	*Bacillus subtilis*	Teichoic acid synthesis	[20]	−6.7
12	Nitroreductase family protein	1YWQ	*Bacillus cereus ATCC 14579*	Reduction of nitrogen-containing compounds	[21]	−4.7
13	HTH-type transcriptional regulator mgrA	2BV6	*Staphylococcus aureus*	Protein biosynthesis	[22]	−5.6
14	Isoleucyl-tRNA synthetase (IleRS)	1QU3	*Staphylococcus aureus*	Protein biosynthesis	[23]	−7.3 *
15	Glutamyl-tRNA(Gln) amidotransferase subunit A	2G5H	*Staphylococcus aureus*	Protein biosynthesis	[24]	−6.1
16	Spore Coat Polysaccharide Biosynthesis Protein SPSA	1H7L	*Bacillus subtilis*	Spore coat biogenesis	[25]	−4.9
17	Teichoic acid biosynthesis protein F	3L7L	*Staphylococcus epidermidis RP62A*	Teichoic acid biosynthesis	[26]	−5.9
18	Transcriptional regulator qacR	1RKW	*Staphylococcus aureus*	Negative regulation of transcription	[27]	−7.2
19	YcgJ protein	2GLU	*Bacillus subtilis*	Methyltransferase activity	To be published	−5.7
20	6-Phosphogluconate Dehydrogenase (Decarboxylating)	2IZ0	*Lactococcus lactis*	Involved in the production of ribulose 5-phosphate, which is used in nucleotide synthesis	[28]	−5.6
**Fungal Protein Targets**						
1	Sterol 14-alpha demethylase (CYP51B)	5FRB	*Aspergillus fumigatus*	Sterol biosynthesis	[29]	−6.8
2	UDP-N-acetylglucosamine pyrophosphorylase	6TN3	*Aspergillus fumigatus Af293*	Cell wall synthesis	[30]	−5
3	*Aspergillus niger* xylanase-I	1T6G	*Aspergillus niger*	Cell wall metabolism	[31]	−5.4
4	Dihydrofolate reductase (DHFR)	4HOF	*Candida albicans*	Metabolite synthesis	[19]	−5.9
5	Aspartic protease	3Q70	*Candida albicans*	Virulence factor	[19]	−5.3
6	N-myristoyltransferase	1IYL	*Candida albicans*	Protein biosynthesis	[19]	−7.2
7	Geranylgeranyltransferase type-1 subunit alpha	3DRA	*Candida albicans*	Metabolite synthesis	[32]	−7.6 *
8	Sterol 14-alpha demethylase (CYP51)	5TZ1	*Candida albicans*	Sterol biosynthesis	[33]	−6.3
9	Glucoamylase-471	1GAH	*Aspergillus awamori*	Involved in the hydrolysis of starch	[34]	−6.8
10	NADPH-dependent D-xylose reductase	1MI3	*Candida tenuis*	Involved in hydrolase activity, hydrolyzing O-glycosyl compounds	[35]	−7.5

* The lowest energy docked conformations did not bind in the reported binding site region. Therefore, these ligands were not considered for further detailed study.

**Table 6:** Ligand–protein interactions analysis for four top-ranked TQ-protein docked systems, namely bacterial Ddl-TQ and qacR-TQ as well as fungal N-myristoyltransferase-TQ and NADPH-dependent D-xylose reductase-TQ complexes.

Enzymes in Complex with TQ	Residues Involved in Hydrophobic Interactions	Residues Involved in H-Bonds	Residues Involved in Pi-Pi Stacking	Residues Involved in Pi-Sigma Interactions
D-alanyl-D-alanine synthetase	Glu57, Glu58, Trp61, Gln64, Glu90, Gln96	Tyr123, Thr89	Tyr93	-
Transcriptional regulator qacR	Val 131, Ile163, Lys190, Ala191, Leu192, Val195, Tyr223	-	Phe151	Phe272
N-myristoyltransferase	His227, Gln226 Tyr335, Leu355, Asn392, Cys393, Leu415, Leu450, Val 449	-	Tyr354	Leu394
NADPH-dependent D-xylose reductase	Asp47, Lys81, His114, Gln191, Ser224, Ile272, Pro273, Lys274, Asn310	Tyr52, Ser218	Tyr217	Trp24

**Table 7:** MMGBSA binding energy in kcal/mol for protein–TQ complexes.

TQ in Complex with:	∆E^VDW^ (van der Waal’s Energy)	∆E^elec^ (Coulombic Energy)	∆G^GB^ (Generalized-Born Polar Solvation Energy)	∆E^SURF^ (Non-Polar Solvation Energy)	∆G^MMGBSA^ (Protein–Ligand Binding Energy)
D-alanyl-D-alanine synthetase	−21.04	−2.73	13.30	−3.12	−13.59 ± 2.65
Transcriptional regulator qacR	−17.84	−6.74	14.98	−3.03	−12.62 ± 4.09
N-myristoyltransferase	−18.35	−3.90	14.26	−2.85	−10.83 ± 3.33
NADPH-dependent D-xylose reductase	−21.41	−2.13	13.68	−3.06	−12.92 ± 2.16

## Data Availability

The manuscript and supplementary data files contain all this research data.

## Supplementary Materials

The following supporting information can be downloaded at https://www.mdpi.com/article/10.3390/antibiotics11010079/s1, Figure S1. FT-IR spectra of TQ, Figure S2. FT-IR spectra of standard TQ, Figure S3. Preliminary antimicrobial activity of TQ showing non-susceptibility among some of the test bacteria; (a) *S. pyogenes* ATCC 19615, (b) *S. pneumoniae* ATCC 49619, (c) *E. faecalis* ATCC 29212, (d) *E. coli* ATCC 25922, (e) *K. pneumoniae* ATCC 27736, (f) *P. aerugenosa* ATCC 9027, (g) *S. typhimurium* ATCC 13311, (h) *S. flexneri* ATCC 12022, (i) *P. mirabilis* ATCC 29906, Figure S4. A 96-well plate is demonstrating the result of MIC for the TQ: Rows A-J showing the tested organisms; (**A**) *S. aureus* ATCC 29213, (**B**) *S. saprophyticus* ATCC 4386, (**C**) MRSA-1, (**D**) MRSA-5, (**E**) MRSA-7, (**F**) *S. epidermidis* ATCC 12228, (**G**) *B. cereus* ATCC 10876, (**H**) *P. vulgaris* ATCC 6380, (**I**) *C. albicans* ATCC 10231, (**J**) *A. niger* ATCC 6275, while columns 1–10 are showing various concentrations of TQ tested in MIC. Column 11 contains suspensions of the test organisms without TQ, which served as a negative control (NC), whereas column 12 contains sterile broth as a sterility control (SC), Figure S5. Overlapping conformations for all four enzyme complexes, namely bacterial; (a) Ddl-TQ and (b) qacR-TQ, and fungal; (c) N-myristoyltransferase-TQ and (d) NADPH-dependent D-xylose reductase-TQ. The conformations at 0 ns are shown as opaque colors, while the conformations at 100 ns are displayed as transparent colors, Figure S6. Snapshots of TQ ligand taken at every 1 ns during 100 ns of MD simulation for bacterial; (a) Ddl-TQ and (b) qacR-TQ, and fungal; (c) N-myristoyltransferase-TQ and (d) NADPH-dependent D-xylose reductase-TQ complexes. The protein conformation has been frozen as 0 ns, Table S1. Antibiotic susceptibility pattern of the tested organisms.
