# Supplementary material for: In Vitro and In Silico Approaches for the Evaluation of Antimicrobial Activity, Time-Kill Kinetics, and Anti-Biofilm Potential of Thymoquinone (2-Methyl-5-propan-2-ylcyclohexa-2,5-diene-1,4-dione) against Selected Human Pathogens
Source: Antibiotics (Basel). 2022 Jan 10;11(1):79. doi: 10.3390/antibiotics11010079 (PMC8773234; doi:10.3390/antibiotics11010079)
Supplement: Supplementary file 1 [file antibiotics-11-00079-s001.zip › antibiotics-1518471-supplementary.pdf]

Article

In-Vitro and *In-Silico* Approaches for the Evaluation of Antimicrobial Activity, Time-Kill Kinetics, and Anti-Biofilm Activity of 2-methyl-5-propan-2-ylcyclohexa-2,5-diene-1,4-dione against Selected Human Pathogens

Kamal A. Qureshi [1,4,*], Mahrukh Imtiaz [2], Adil Parvez [3], Pankaj K. Rai [4], Mariusz Jaremko [5], Abdul-Hamid Emwas [6], Avinash D. Bholay [7], M. Qaiser Fatmi [2,*]

[1] Department of Pharmaceutics, Unaizah College of Pharmacy, Qassim University, Unaizah, 51911, Saudi Arabia
[2] Department of Biosciences, COMSATS University Islamabad, Islamabad, 45600, Pakistan; mahrukhimtiaz92@yahoo.com
[3] Department of Biotechnology, School of Chemical and Life Sciences, Jamia Hamdard University, New Delhi, 110062, India; adilparvez.92@gmail.com
[4] Department of Biotechnology, Faculty of Biosciences, Invertis University, Bareilly, 243123, India; pankaj.r@invertis.org
[5] Biological and Environmental Sciences and Engineering Division (BESE), King Abdullah University of Science and Technology (KAUST), Thuwal, 23955-6900, Saudi Arabia; mariusz.jaremko@kaust.edu.sa
[6] Core Labs, King Abdullah University of Science and Technology (KAUST), Thuwal, 23955-6900, Saudi Arabia; abdelhamid.emwas@kaust.edu.sa
[7] Department of Microbiology, KTHM College, Savitribai Phule Pune University (SPPU), Nashik, 422002, India

* Correspondence: ka.qurishe@qu.edu.sa (K.A.Q.); qaiser.fatmi@comsats.edu.pk (M.Q.F.)

Supplementary Information

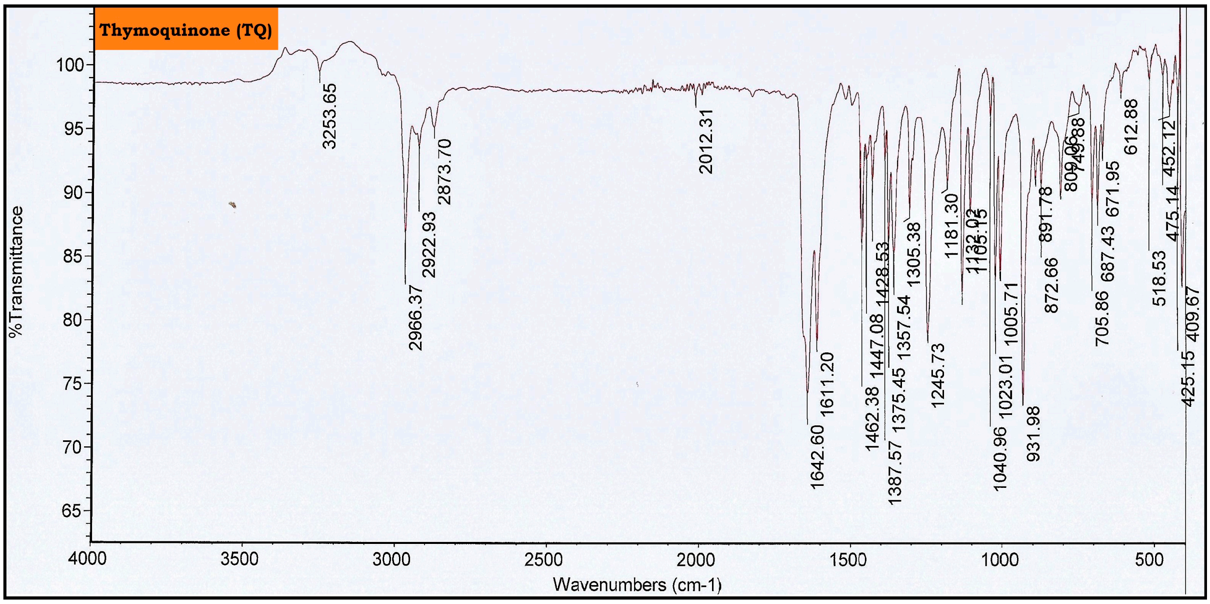

Figure S1. FT-IR spectra of TQ.

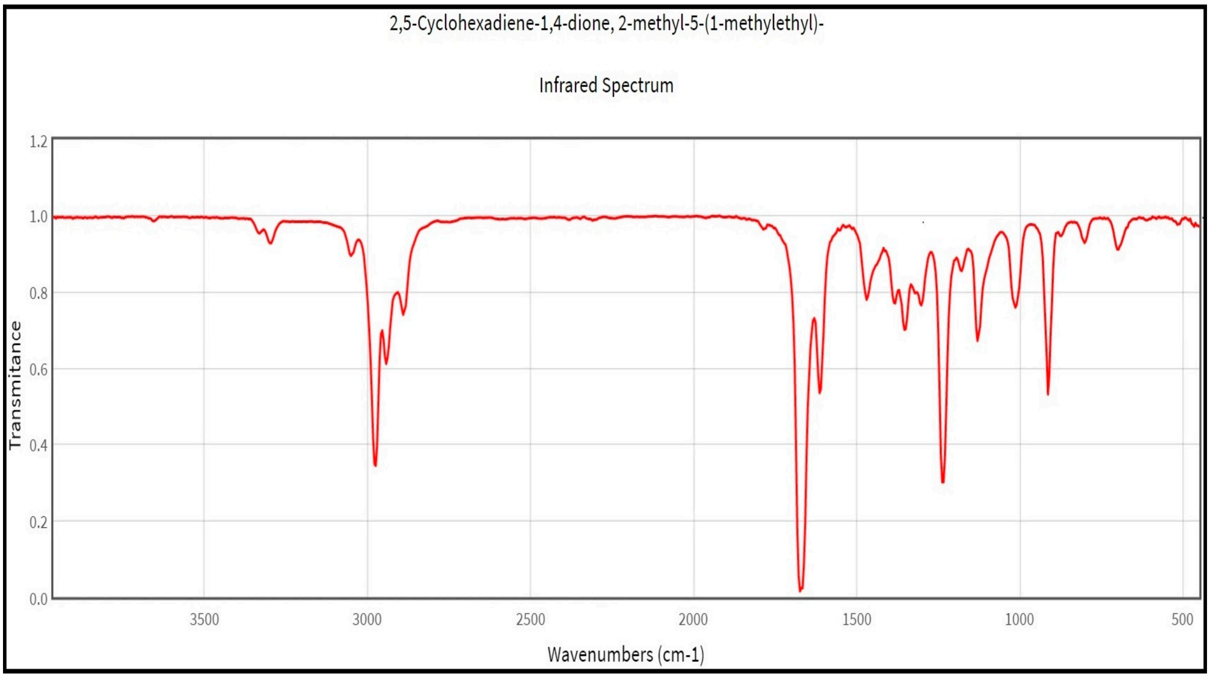

Figure S2. FT-IR spectra of standard TQ

(Source: https://webbook.nist.gov/cgi/cbook.cgi?ID=C490915&Mask=80)

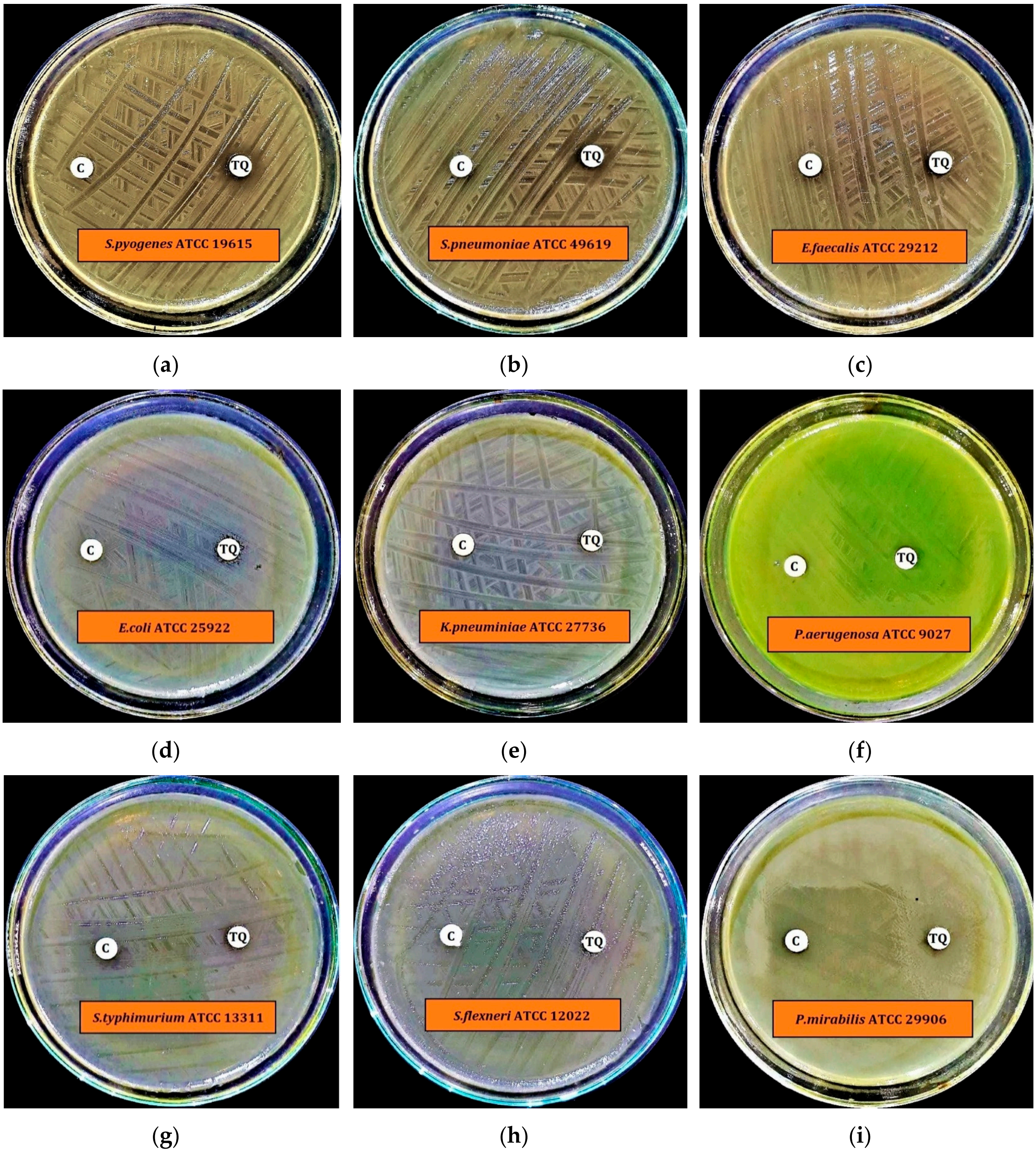

Figure S3. Preliminary antimicrobial activity of TQ showing non-susceptibility among some of the test bacteria; (**a**) *S. pyogenes* ATCC 19615, (**b**) *S. pneumoniae* ATCC 49619, (**c**) *E. faecalis* ATCC 29212, (**d**) *E. coli* ATCC 25922, (**e**) *K. pneumoniae* ATCC 27736, (**f**) *P. aerugenosa* ATCC 9027, (**g**) *S. typhimurium* ATCC 13311, (**h**) *S. flexneri* ATCC 12022, (**i**) *P. mirabilis* ATCC 29906.

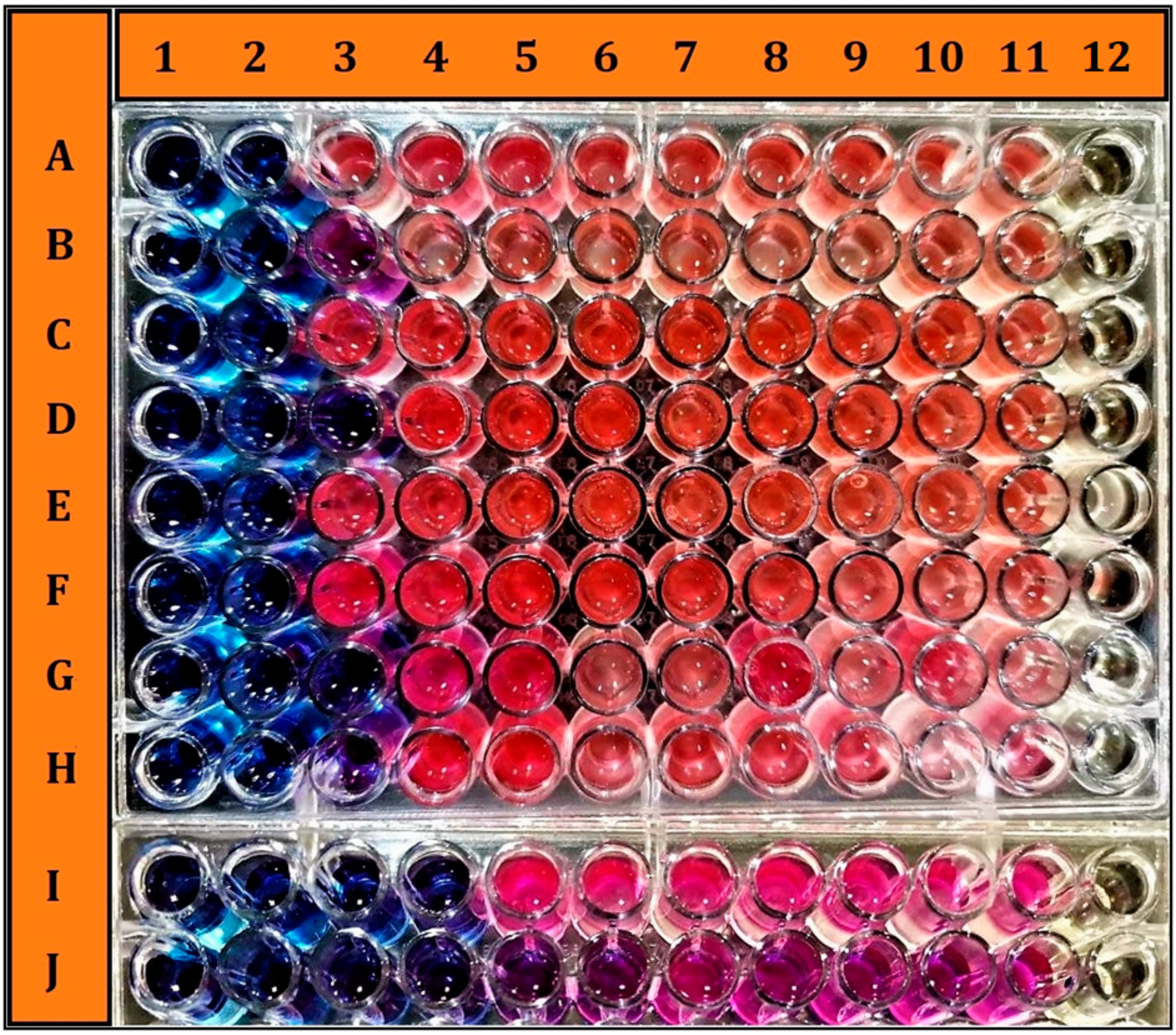

Figure S4. A 96-well plate is demonstrating the result of MIC for the TQ: Rows A-J showing the tested organisms; (**A**) *S. aureus* ATCC 29213, (**B**) *S. saprophyticus* ATCC 4386, (**C**) MRSA-1, (**D**) MRSA-5, (**E**) MRSA-7, (**F**) *S. epidermidis* ATCC 12228, (**G**) *B. cereus* ATCC 10876, (**H**) *P. vulgaris* ATCC 6380, (**I**) *C. albicans* ATCC 10231, (**J**) *A. niger* ATCC 6275, while columns 1-10 are showing various concentrations of TQ tested in MIC. Column 11 contains suspensions of the test organisms without TQ, which served as a negative control (NC), whereas column 12 contains sterile broth as a sterility control (SC).

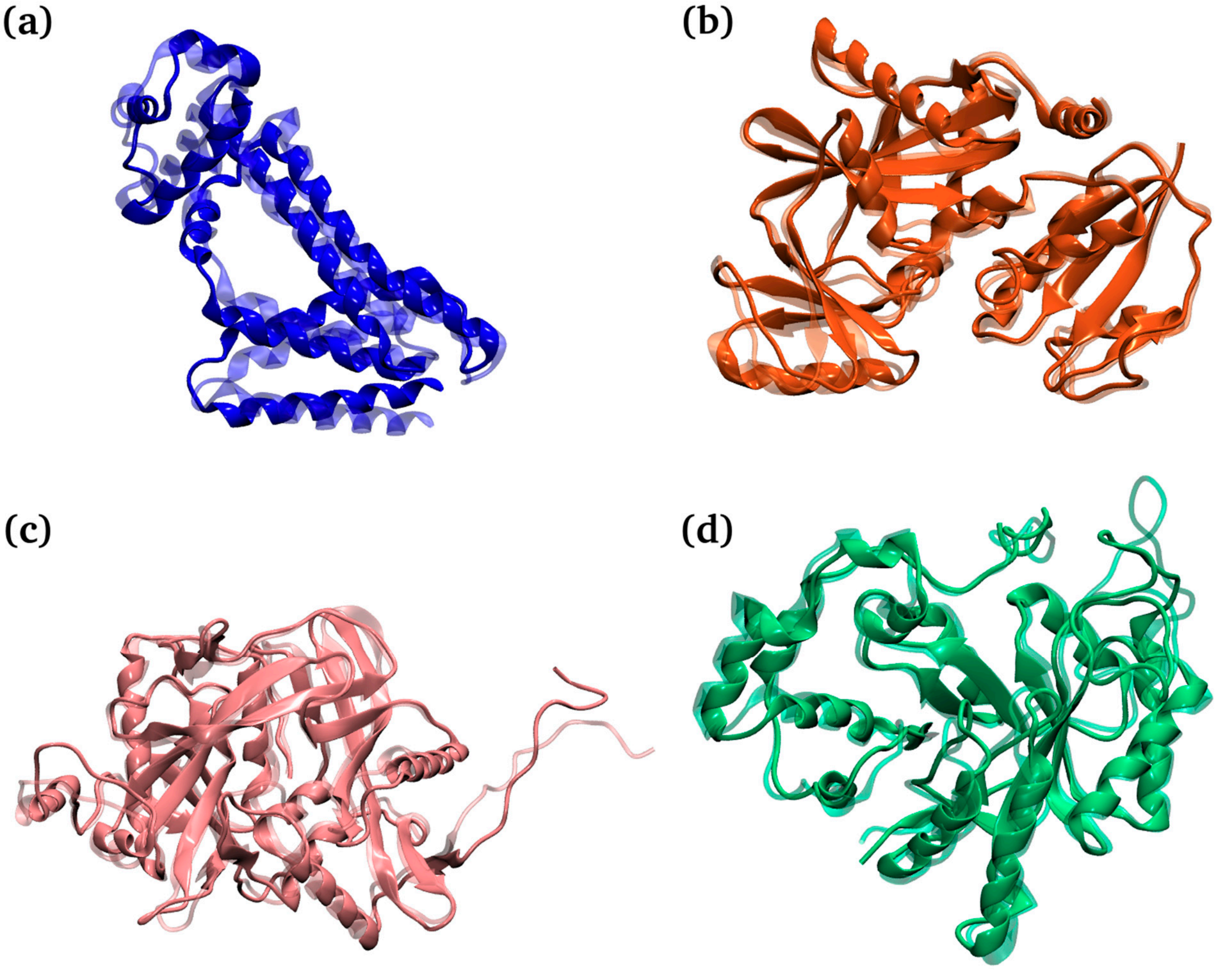

Figure S5. Overlapping conformations for all four enzyme complexes, namely bacterial; (a) Ddl-TQ and (b) qacR-TQ, and fungal; (c) N-myristoyltransferase-TQ and (d) NADPH-dependent D-xylose reductase-TQ. The conformations at 0 ns are shown as opaque colors, while the conformations at 100 ns are displayed as transparent colors.

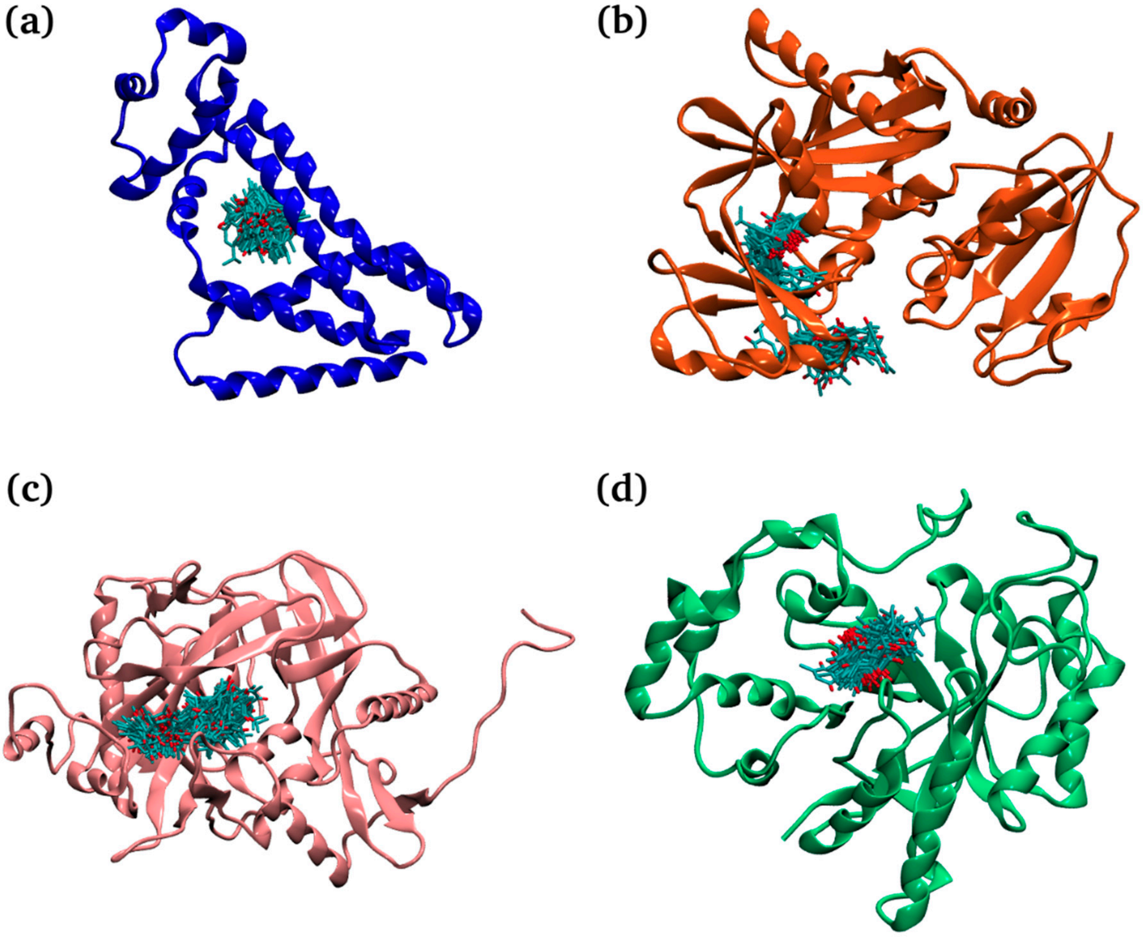

Figure S6. Snapshots of TQ ligand taken at every 1 ns during 100 ns of MD simulation for bacterial; (a) Ddl-TQ and (b) qacR-TQ, and fungal; (c) N-myristoyltransferase-TQ and (d) NADPH-dependent D-xylose reductase-TQ complexes. The protein conformation has been frozen as 0 ns.

Table S1. Antibiotic susceptibility pattern of the tested organisms

Test organisms	Antibiotics (µg) v/s Zone of Inhibition Diameter (mm)									
	**P (2 µg)**	**E (10 µg)**	**AMP (30 µg)**	**AML (25 µg)**	**AMC (30 µg)**	**CTX (30 µg)**	**CN (10 µg)**	**C (30 µg)**	**TE (30 µg)**	**IMP (10 µg)**
S. aureus ATCC 29213	6.0 **R**	18.8 **S**	8.7 **R**	11.7 **R**	20.6 **S**	6.0 **R**	NT	NT	NT	NT
S. saprophyticus ATCC 4386	9.9 **R**	20.7 **S**	13.5 **R**	22.2 **S**	25.0 **S**	6.0 **R**	NT	NT	NT	NT
MRSA-1	6.0 **R**	17.5 **S**	6.0 **R**	6.0 **R**	12.8 **R**	6.0 **R**	NT	NT	NT	NT
MRSA-5	6.0 **R**	19.1 **S**	6.0 **R**	6.7 **R**	10.5 **R**	6.0 **R**	NT	NT	NT	NT
MRSA-7	6.0 **R**	18.8 **S**	6.0 **R**	6.7 **R**	11.5 **R**	6.0 **R**	NT	NT	NT	NT
S. epidermidis ATCC 12228	6.0 **R**	17.1 **S**	6.0 **R**	6.0 **R**	14.1 **R**	6.0 **R**	NT	NT	NT	NT
B. cereus ATCC 10876	6.0 **R**	25.5 **S**	9.2 **R**	17.3 **S**	25.7 **S**	6.0 **R**	NT	NT	NT	NT
S. pyogenes ATCC 19615	6.0 **R**	14.0 **R**	14.5 **R**	19.8 **S**	23.5 **S**	6.0 **R**	NT	NT	NT	NT
S. pneumoniae ATCC 49619	6.0 **R**	14.7 **R**	10.1 **R**	20.6 **S**	21.1 **S**	6.0 **R**	NT	NT	NT	NT
E. faecalis ATCC 29212	6.0 **R**	15.7 **R**	10.4 **R**	20.5 **S**	23.1 **S**	6.0 **R**	NT	NT	NT	NT
E. coli ATCC 25922	NT	NT	NT	NT	16.6 **R**	24.0 **S**	16.5 **R**	20.0 **S**	18.6 **S**	22.2 **S**
K. pneumoniae ATCC 27736	NT	NT	NT	NT	15.0 **R**	20.0 **S**	15.0 **R**	20.3 **S**	14.9 **R**	22.1 **S**
P. aerugenosa ATCC 9027	NT	NT	NT	NT	6.0 **R**	12.2 **R**	6.0 **R**	14.2 **R**	6.0 **R**	19.7 **S**
S. typhimurium ATCC 13311	NT	NT	NT	NT	18.8 **S**	21.4 **S**	15.9 **R**	22.4 **S**	17.2 **S**	19.2 **S**
S. flexneri ATCC 12022	NT	NT	NT	NT	17.5 **S**	25.8 **S**	13.2 **R**	17.0 **S**	17.0 **S**	23.2 **S**
P. vulgaris ATCC 6380	NT	NT	NT	NT	20.9 **S**	22.8 **S**	17.6 **S**	15.6 **R**	7.8 **R**	20.9 **S**
P. mirabilis ATCC 29906	NT	NT	NT	NT	20.0 **S**	23.2 **S**	17.6 **S**	6.0 **R**	6.0 **R**	12.1 **R**

Note: ≥17.0 mm zone of inhibition diameter= Susceptible (**S**), <17.0 mm zone of inhibition diameter= Resistant (**R**). **P=** Penicillin; **E=** Erythromycin; **AMP=** Ampicillin; **AML=** Amoxicillin; **AMC=** Amoxycillin/clavulanic acid; **CTX=** Cefotaxime; **CN=** Gentamycin; **C=** Chloramphenicol; **TE=** Tetracycline; **IMP=** Imipinem; **NT=** Not tested.
